# Stewarding the future: leadership, innovation, and the next chapter of healthcare-associated infections and antimicrobial resistance prevention

**DOI:** 10.1017/ash.2026.10394

**Published:** 2026-05-11

**Authors:** Lisa L. Maragakis

**Affiliations:** Department of Medicine, Division of Infectious Diseases, Johns Hopkins University School of Medicine, Baltimore, USA

## Introduction

Lisa Maragakis, M.D., M.P.H., FSHEA, FIDSA, is a professor of medicine and epidemiology at Johns Hopkins University School of Medicine and the Johns Hopkins Bloomberg School of Public Health. Dr. Maragakis is the Senior Director of Infection Prevention for The Johns Hopkins Health System and the Executive Director of the Johns Hopkins Hospital Biocontainment Unit. She previously served as Co-Chair for the 2022 Update of the Compendium of Strategies to Prevent Healthcare-Associated Infections and as Co-Chair of the Centers for Disease Control and Prevention’s Healthcare Infection Control Practices Advisory Committee (HICPAC). She is the 2026–2027 President of the Board of Trustees of the Society for Healthcare Epidemiology of America (SHEA).

In this moment of complexity and opportunity in health care, professional societies play a critical role. In this conversation with Antimicrobial Stewardship and Healthcare Epidemiology, Dr. Lisa Maragakis reflects on her strategic vision for the organization, the evolving challenges of the field, and the leadership principles guiding her tenure. The discussion explores innovation, advocacy, workforce development, and what it will take to steward the field into its next era.

For more, visit SHEA podcast—interview with Dr. Lisa Maragakis

## You assume the presidency at a time of continued strain on healthcare systems and rising antimicrobial resistance. What do you see as the defining challenge—or opportunity—for SHEA right now?

That is a difficult question because there are so many competing challenges one might name. I would start by saying that I am very proud that our SHEA members are well versed in responding to challenges. It is one of our strengths and an area of expertise for our members. We have demonstrated this time and again over the last several years through resilience and resourcefulness.

I would also emphasize that challenges are opportunities. It is easy to get bogged down in enumerating all the negatives associated with the challenges an individual or organization faces, but they really are opportunities.

So, what is the main challenge we face? It is the unprecedented dismantling of our federal public health infrastructure. Given what we do—protecting patients from healthcare-associated infections, emerging antimicrobial resistance, and the overuse of antimicrobials—we rely on and are deeply embedded in the public health infrastructure.

To have that infrastructure dismantled, underfunded, and losing expertise is a major challenge. At the same time, this challenge creates an opportunity for SHEA to step forward into the widening gap and provide evidence-based information to the public, our patients, and our colleagues. This is the work we already do. It may make the work harder, but in some ways, it is also an opportunity for SHEA to grow our membership, partnerships, skills, and influence.

We need to reach out to our members, recruit new members, and partner with other organizations to bring a diversity of perspectives into our work so we can address these challenges together.

## Looking beyond the immediate term, what structural or cultural shifts would you like to see SHEA catalyze over the next 5–10 years?

That is another great question, and it relates closely to my previous answer. SHEA is currently constructing our next five-year strategic plan, so it is a great moment to think about the future.

We want to look beyond the challenges immediately in front of us and ask: where do we want to be in five years, and how do we position ourselves for the future?

Strengthening partnerships will be a critical piece of that strategic plan. Building redundancy and resilience—concepts I mentioned earlier—will help us respond more effectively to future challenges.

Another priority is growing our membership and bringing in diverse voices. We want more members and partners who can find a home in SHEA and contribute to the significant amount of work required to address challenges in a changing public health infrastructure.

That includes strengthening connections with public health partners at the state, local, and federal levels. We also need to continue emphasizing that while we are focused on infection prevention and healthcare epidemiology, we are equally committed to antimicrobial stewardship.

There are also emerging challenges related to sustainability and global health. There is so much work to do that we really need to keep growing.

Finally, we must think and act globally. We are the Society for Healthcare Epidemiology of America, but we intend to emphasize and continue growing our membership and collaborations with international colleagues as well.

## Healthcare epidemiology leaders frequently navigate tensions between evidence, operational realities, and institutional priorities. How do you approach decision-making when consensus is challenging?

That situation is always challenging. It is a daily question for me and most SHEA members.

First, we must always bring the evidence to the table. Our members are at the forefront of generating the evidence that informs our field. One current challenge is ensuring continued advocacy for funding so that research can continue and we have the evidence base needed to guide decisions and priorities.

At the same time, we must acknowledge operational realities. One of the most difficult aspects of our work is making the financial case for prevention.

We need to develop skills and continue to improve our ability to articulate the return on investment—why resources should be devoted to infection prevention and antimicrobial stewardship, and how those investments benefit both patient safety and the financial health of healthcare institutions.

This is a critical skill, and SHEA is well positioned to provide our members with the information they need to make that case to organizations that are facing significant financial pressures.

## We are witnessing rapid growth in areas such as genomic epidemiology, predictive analytics, and artificial intelligence (AI)-enabled surveillance. What innovations most excite you—and what guardrails are necessary to ensure responsible implementation?

This is a fascinating question that applies not only to our field but to many others.

One area that excites me most is the potential of predictive analytics and AI to inform the data we use to drive interventions. In infection prevention and antimicrobial stewardship, we know we can change what we measure. Technology and AI allow us to measure more things more accurately, and to analyze data more effectively.

This can provide deeper insights and allow us to share those insights in ways that improve practice.

One example is our ability to look more closely at process measures, not just outcomes. At my institution, we are implementing a pilot program using visualization and AI in operating rooms to collect additional data about what occurs in that complex environment.

That example highlights both the potential and the challenges of these technologies. On the one hand, we can gather much richer data and potentially identify opportunities to improve patient safety. On the other hand, it raises important questions about privacy, how data are used, how they are analyzed, and how accountability is handled for both patients and clinicians.

Another guardrail is ensuring that human expertise remains central. These technologies should support—not replace—the expertise of infection prevention and stewardship professionals. Much of our work depends on relationships, influence, and understanding what is really happening on the ground.

Technology can enhance that work, but it cannot replace it.

## Professional societies increasingly serve as policy voices. Where do you believe SHEA can have the greatest impact in shaping national or global infection prevention and antimicrobial stewardship policy?

Our members have deep expertise and experience in both healthcare epidemiology and antimicrobial stewardship, so we absolutely should be at the table in policy discussions. In fact, many of our members are already leading nationally and internationally in these areas.

Returning to our earlier discussion about the challenges facing public health infrastructure, this moment creates an opportunity for SHEA to contribute even more actively to policy development.

We have long been involved in creating guidelines, but we can also play an important role in shaping broader national and international policy around issues such as multidrug-resistant organisms, global antimicrobial use, and sustainability.

These are complex, intersecting issues, and I hope our members will continue to lead and participate actively in policy development.

## The infectious diseases and healthcare epidemiology workforce pipeline remains fragile. What concrete steps can societies like SHEA take to cultivate and sustain the next generation of leaders?

This is a critical question. It may feel especially acute right now, but the challenge is not new.

Healthcare epidemiology and antimicrobial stewardship have evolved rapidly over a relatively short period of time. These fields are growing, and the demands continue to increase.

Even as we work to ensure that a new generation is coming forward to take on this work, we also need more people overall who value and are trained in this field.

As an organization, we need to be strong mentors. We need to communicate clearly why this work matters and why it is exciting and rewarding. We also need to think more broadly about the pathways that can lead people into this work.

That means reaching beyond traditional training pathways and engaging individuals from other disciplines whose interests align with our goals.

Ultimately, this connects back to the themes we discussed earlier—expanding membership, broadening partnerships, and bringing diverse perspectives together to address complex challenges like antimicrobial resistance and healthcare-associated infections.

## When your presidency concludes, what would success look like—for you and for SHEA?

One year is a relatively short period of time, so everything is somewhat relative.

I view this role as a stewardship of the association. Success means continuing to build toward the future.

Developing our strategic plan is a central part of that work. It will serve as a roadmap for the organization. As we discussed earlier, we are living in unprecedented times with enormous challenges.

Now more than ever, we need a strategic plan that helps guide us beyond the present moment and keeps us focused on the future. That includes adapting to shifts in technology, workforce dynamics, and evolving public health challenges.

We may not solve everything during this time, but success would mean putting the right building blocks in place so that the next president—and the presidents after that—along with our members and board of trustees, feel prepared to meet the future.

